# Toxicity spectrum of pegvaliase: A pharmacovigilance analysis using the FAERS database

**DOI:** 10.1186/s13023-025-03864-4

**Published:** 2025-06-20

**Authors:** Luyao Xu, Kaili Mao, Songyang Zhong, Huayu Sun, Hongliang Zheng, Zhenling Fu

**Affiliations:** Department of Pharmacy, The Quzhou Affiliated Hospital of Wenzhou Medical University, Quzhou People’s Hospital, No.100 Minjiang Road, Kecheng District, Quzhou, 324000 Zhejiang China

**Keywords:** Pegvaliase, Pharmacovigilance, Data mining, FAERS database, Adverse event

## Abstract

**Objective:**

This research aimed to assess the safety of pegvaliase through analyzed the data from the FAERS database, thus providing a theoretical foundation for the rational and safe application of pegvaliase in clinical settings.

**Methods:**

Pegvaliase-associated adverse event reports were searched in FAERS database from the 2018 Q3 to 2023 Q2. These data were further mined through Four different algorithms, including ROR, PRR, BCPNN, and EBGM.

**Results:**

A total of 5,076 AEs reports were obtained from the FAERS database. At the PTs level, it was discovered that AE reports associated with pegvaliase as the primary suspect were connected to the 27 SOCs. Among these PTs, 83 signals in total were found, and each of them concurrently complied with the four algorithms. Further, we ranked PTs first for arthralgia in order of frequency and first for decreased amino acid levels in order of signal intensity. The median time to onset of adverse reactions was 15 days.

**Conclusion:**

We mined and analyzed the AE signals of pegvaliase based on the FAERS database, it turned out that they were generally consistent with the drug inserts and clinical trial results. However, potential new AE signals were revealed, providing a basis for the identification of adverse reactions in the clinical setting.

**Supplementary Information:**

The online version contains supplementary material available at 10.1186/s13023-025-03864-4.

## Introduction

Phenylketonuria (PKU) is an autosomal recessive disorder caused by a deficiency of the phenyalanine hydroxylase (PAH), which leads to elevated levels of phenylalanine (Phe) and affects approximately 1 in 24,000 people, with an estimated 0.45 million affected individuals worldwide [1]. Hyperphenylalaninemia (HPA), characterized by elevated levels of Phe in the blood and cerebrospinal fluid, is caused by dysregulated Phe metabolism. Tetrahydrobiopterin (BH4), a pivotal cofactor in Phe metabolism, is essential for the enzymatic breakdown of Phe [2]. PKU, the main type of HPA, has phe levels exceeding 360 µmol/L [3]. The absence of PAH eventually causes elevated levels of Phe in the blood and brain, which can cause intellectual incapacity, epilepsy, seizures, microcephaly, delayed speech, psychotic movement disorders, eczema, generalized hypopigmentation of skin (including eyes and hair), and a musty sweat odor if PKU is left untreated [4–8]. Most cases of PKU are detected shortly through newborn screening (NBS) shortly after birth. Early and sustained management of Phe levels improves health-related quality of life in these patients [9]. As a result, severe PKU symptoms and indicators are uncommon [10]. Phe levels should be kept between 120 and 360 µmol/L throughout life, according to American College of Medical Genetics and Genomics (ACMG) standards, for the best possible outcome [7]. The most common PKU treatment strategy, known as a restricted Phe diet, entails cutting back on natural protein sources and replacing them with low- or Phe-free alternatives as well as customized low-protein meals (SLPFs). However, Long-term adherence to the restrictive diet is challenging for patients [11]. Sapropterin was approved by Food and Drug Administration (FDA) on December 13, 2007, and was the first pharmacologic therapy for treatment of PKU. Sapropterin is used in conjunction with a phenylalanine-restricted diet. The efficacy of sapropterin depends on the presence of residual PAH enzyme activity, which works for approximately 25–50% of PKU patients [12]. The new therapy option of Pegvaliase is of particular importance as it has brought new hope to patients who do not respond to dietary restrictions and sapropterin, filling a critical gap in treatment options for this patient population.

Pegvaliase (Palynziq^®^, BioMarin Pharmaceutical Inc., Novato, CA, USA) was authorized as an enzyme-substitution therapy for adults (US) or patients aged ≥ 16 years (Europe) with PKU and uncontrolled blood Phe concentrations > 600µmol/L on current management [13, 14]. According to Janet Thomas et al., Of the 261 adult participants who received an average of 36.6 months of pegavarase treatment, 71.3%, 65.1%, and 59.4% achieved clinically significant blood Phe levels of ≤ 600, ≤360, and ≤ 120 µmol/L, respectively [15, 16]. Phase 1 to phase 3 clinical trial data indicates significant drops in blood phenylalanine concentrations and amelioration of inattention and mood symptoms when pegvaliase is used [17–19]. In the Phase 3 PRISM-1 and PRISM-2 clinical trials [15, 19], pegvaliase-induced blood Phe reductions were clinically meaningful and statistically significant versus placebo and were sustained at a population level at 24 months of follow-up. As pegvaliase becomes more increasingly used, the attention to its associated adverse reactions and drug-related adverse events, such as anaphylaxis, injection site response, and arthralgia, has also increased. To guarantee patient safety and maximize treatment results, it is crucial to focus on these adverse occurrences. It allows the creation of monitoring and preventive measures, helps healthcare providers manage medications, and enables individualized treatment programs. In the end, these initiatives help to improve patient outcomes, optimize treatment plans, and advance the medical industry.

The FDA Adverse Event Reporting System (FAERS) database is a publicly available, online-accessible database of reports from the FDA’s self-reporting system. It has been widely used in the research field of pharmacovigilance [20, 21]. This research aimed to assess the safety of pegvaliase through the analysis of data from FAERS using data mining techniques, thus providing a point of reference for clinical monitoring and risk identification.

## Materials and methods

### Data sources and mining

**Fig. 1:**
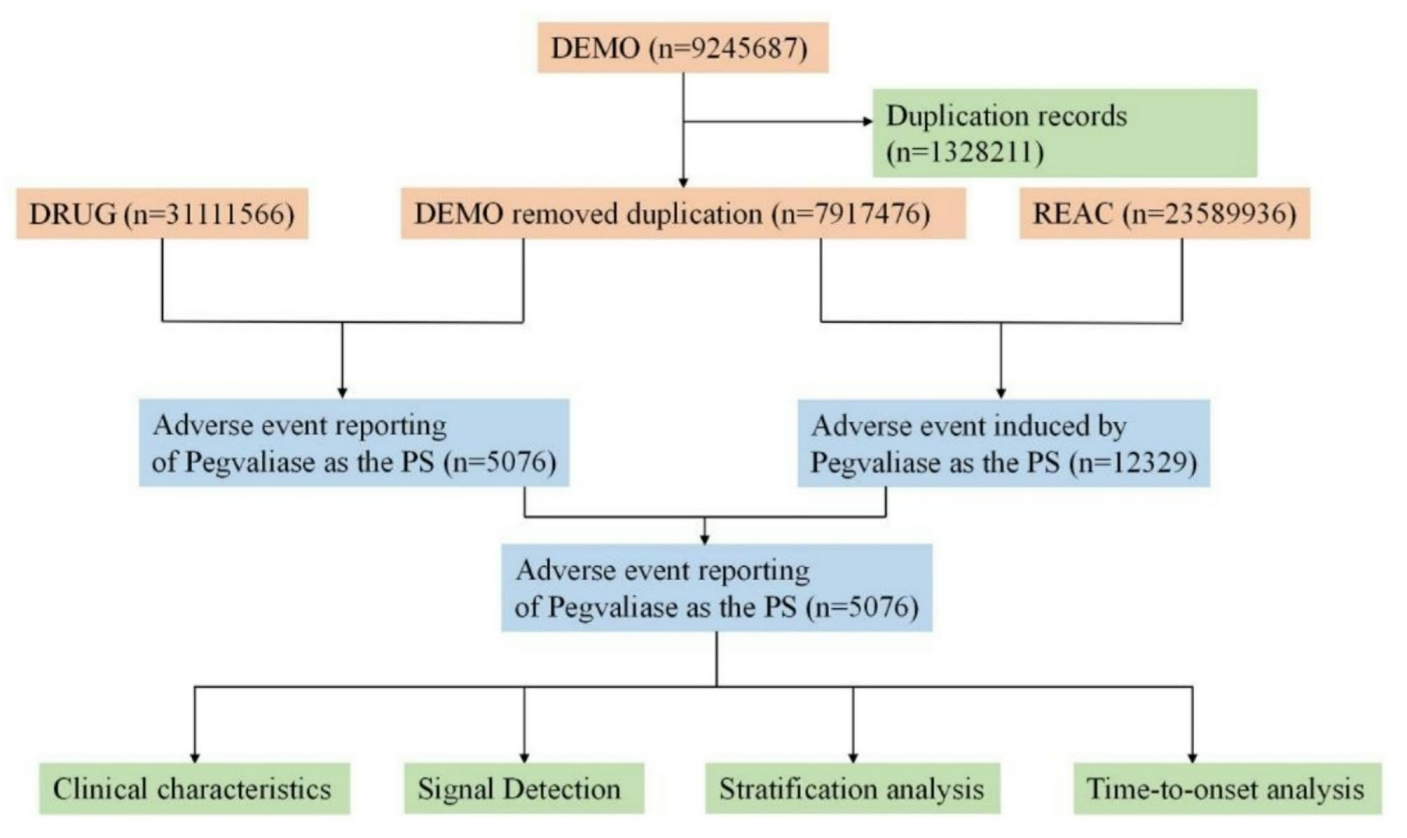
FDA FAERS Database Mining Workflow Diagram

We launched a retrospective pharmacovigilance study on adverse events associated with pegvaliase. We extract all pegvaliase-related adverse events (AEs) from 2018 Q3 to 2023 Q2 from the FAERS database. A flow diagram of database mining was showed in Fig. 1. During the study period, a total of 19,806,910 reports of pegvaliase were obtained from the FAERS database. Duplicated records were deleted according to the deduplication process recommended by FDA. We selected PRIMARYID, CASEID, and FDA_DT fields in the demographics table and sorted them in the order of CASEID, FDA_DT, and PRIMARYID. For reports with the same CASEID, we retained the one with the largest FDA_DT value. For reports with the same CASEID and FDA_DT, we reserved the one with the largest PRIMARYID value. Drugs were categorized into four patterns: PS (primary suspect), SS (secondary suspect), C (concomitant), and I (interacting). In this study, we only analyzed monotherapy and incorporated the reports only identified as the PS drug leading to AEs to obtain better signal intensity. The FAERS database consisted of seven data files: patient demographic information (DEMO), drug information (DRUG), adverse event information (REAC), patient outcome information (OUTC), report source information (RPSR), drug therapy date information (THER), and drug indication (INDI) [22]. The data was retrieved using the keywords “pegvaliase/palynziq/pegvaliase-pqpz” for collecting patient demographics, drug information, AE information, patient outcomes and so on to further statistically analyze.

The System organ class (SOC), High Level Group Term (HLGT), High Level Term (HLT), Preferred term (PT), and Lowest Level Term (LLT) comprise the hierarchical structure of the MedDRA nomenclature [23]. All valid pegvaliase-related AEs in our study were coded using preferred terms (PTs) and grouped into the System Organ Class (SOC) according to the Medical Dictionary for Regulatory Activities (MedDRA) version 26.1.

### Statistical analysis

The proportional disequilibrium method and Bayesian method are critical analytical tools utilized in the field of pharmacovigilance. The proportional disequilibrium method involves a comparison of the occurrence proportions of adverse events between a target drug and all other drugs. Both the proportional reported odds ratio (PRR) and the reported odds ratio (ROR) are included in this analysis [21]. On the other hand, the Bayesian method incorporates two prominent algorithms: the Bayesian confidence propagation neural network (BCPNN) and the multiple Gamma Poisson reduction method (MGPS) [24]. In order to gain more reliable results, this study adopted four distinct algorithms: ROR, PRR, BCPNN, and MGPS. Table 1 shows the four-cell table of the ratio imbalance method. The four algorithms were summarized in Table 2, which were used to quantify the signal strength value representing the relevance between the interested drug and specific AE. If the criteria listed in Table 2 were met simultaneously, an AE would be considered highly associated with the treatment of the interested drug, with higher values indicating a more robust statistical correlation. Data regarding gender, age, reported year, reporter, geographical distribution, outcome of AEs weredisplayed using descriptive statistics and the count, data were expressed as frequency(%). Moreover, time-to-onset data that was defined as the period of time from the initiation of the use of pegvaliase to the onset of AEs were analyzed using the Weibull shape parameter (WSP) test. The incidence of AEs after the initiation of treatment depends on the drug mechanism of action and often varies over time. In contrast AEs that are not associated with drug treatment occur at aconstant rate. The WSP test can determine the varying ratio of incidence of AEs. All analyses were performed using Rsoftware version 4.3.1(The R Foundation for Statistical. Computing, Vienna. Austria).

**Table 1:** Four-cell table of proportional disequilibrium method

Drugs	Target adverse reaction	Other adverse reactions	Total
Target drug	a	b	a + b
Other drugs	c	d	c + d
Total	a + c	b + d	n = a + b + c + d

a: the number of reports containing both the suspect drug and the suspect adverse drug reaction; b: the number of reports containing the suspect adverse drug reaction with other medications (except the drug of interest); c: the number of reports containing the suspect drug with other adverse drug reactions (except the event of interest); d: the number of reports containing other medications and other adverse drug reactions; n: the number of all reports

**Table 2:** Four major algorithms used for signal detection

Algorithms	Equation	Criteria
ROR	ROR = ad/b/c	lower limit of 95% CI > 1, *N* ≥ 3
	95%CI = e^ln(ROR)±1.96(1/a+1/b+1/c+1/d)^0.5^	
PRR	PRR = a(c + d)/c/(a + b)	PRR ≥ 2, χ^2^ ≥ 4, *N* ≥ 3
	χ^2^=[(ad-bc)^2](a + b + c + d)/[(a + b)(c + d)(a + c)(b + d)]	
BCPNN	IC = log_2_a(a + b + c + d)(a + c)(a + b)	IC025 > 0
	95%CI = E(IC) ± 2 V(IC)^0.5	
EBGM	EBGM = a(a + b + c + d)/(a + c)/(a + b)	EBGM05 > 2
	95%CI = e^ln(EBGM)±1.96(1/a+1/b+1/c+1/d)^0.5^	

Equation: a, number of reports containing both the target drug and target adverse drug reaction; b, number of reports containing other adverse drug reaction of the target drug; c, number of reports containing the target adverse drug reaction of other drugs; d, number of reports containing other drugs and other adverse drug reactions. 95%CI, 95% confidence interval; N, the number of reports; χ^2^, chi-squared; IC, information component; IC025, the lower limit of 95% CI of the IC; E(IC), the IC expectations; V(IC), the variance of IC; EBGM, empirical Bayesian geometric mean; EBGM05, the lower limit of 95% CI of EBGM

## Results

### General characteristics

Following a sequence of data processing steps, 5,076 adverse event reports of pegvaliase as the PS were acquired from 2018 Q3 to 2023 Q2. The detailed data processing procedure is shown in Fig. 1.The characteristics of the AE reports that were turned in for pegvaliase are shown in Table 3. The proportion of females (41.2%) was higher than that of males (27.4%), with 31.4% of unknown sex cases. Patients between the ages of 18 and 45 accounted for the highest proportions of reports, comprising 34.12%. Furthermore, with 58.2% (*n* = 2953) of the age group that was not identified, it also represented a sizeable amount. Following the medicine’s debut in 2018, the number of reported AEs had progressively increased, peaked in 2020 (23.1%), and then gradually dropped thereafter. Most of the reports were submitted by consumers (83.0%), followed by physicians (3.9%), pharmacists (1.0%), other health professionals (0.7%), and not specified people (11.3%). America reported the highest percentage (98.2%), followed by Germany (1.5%) and Italy (0.2%). The most commonly reported severe outcome was other serious medical event with 571 cases (11.1%), followed by hospitalization with 151 cases (2.9%), life-threatening situations with 40 cases (0.8%), death with 6 cases (0.1%), and disability with 2 cases (0.0%). From Table 3 of the database, we extracted the times at which adverse events pertaining to pegvaliase occurred. However, 2759 (54.4%) of the cases did not report the onset time of AEs. The median start time was 15 days with an interquartile range (IQR) of 1 to 2,372 days. The majority of AEs happened in the initial month (*n* = 1591, 31.3%), followed by 31–60 days (*n* = 322, 6.3%), 61–90 days(2.0%). There were also cases reported between more than 360 days (*n* = 94, 1.9%), 91–120 days (*n* = 76, 1.5%), 181–360 days (*n* = 74, 1.5%), 151–180 days (*n* = 34, 0.7%), and 121–150 days (*n* = 23, 0.5%) following the start of pegvaliase. It was noteworthy that our findings suggested that adverse events were most likely to occur in the early stages.

**Table 3:** Clinical characteristics of reports with Pegvaliase from the FAERS database (2018 Q3 to 2023 Q2)

Characteristics	Case Number, *n*	Case Proportion, %
**Gender**		
Female	2090	41.2
Male	1392	27.4
Not Specified	1594	31.4
**Age(Years)**		
< 18	84	1.7
≥ 18, <45	2032	40.0
≥ 45, <65	7	0.1
Not Specified	2953	58.2
**Reported year**		
2023	764	15.1
2022	887	17.5
2021	1136	22.4
2020	1173	23.1
2019	971	19.1
2018	145	2.9
**Reporters**		
Consumer	4214	83.0
Not Specified	576	11.3
Physician	197	3.9
Other health-professional	38	0.7
Pharmacist	51	1.0
**Report countries**		
Argentina	2	0.0
Not Specified	1	0.0
Germany	75	1.5
United Kingdom	1	0.0
Italy	9	0.2
Japan	1	0.0
Kuwait	2	0.0
Russia	1	0.0
The United States	4984	98.2
**Outcomes**		
Death	6	0.1
Disability	2	0.0
Hospitalization-Initial or Prolonged	151	2.9
Life-Threatening	40	0.8
Other Serious (Important Medical Event)	571	11.1
Not Specified	4364	85.0
Time to onset		
0–30 d	1591	31.3
121–150 d	23	0.5
151–180 d	34	0.7
181–360 d	74	1.5
31–60 d	322	6.3
61–90 d	103	2.0
91–120 d	76	1.5
> 360 d	94	1.9
Not Specified or missing	2759	54.4

### Signal detection

**Fig. 2:**
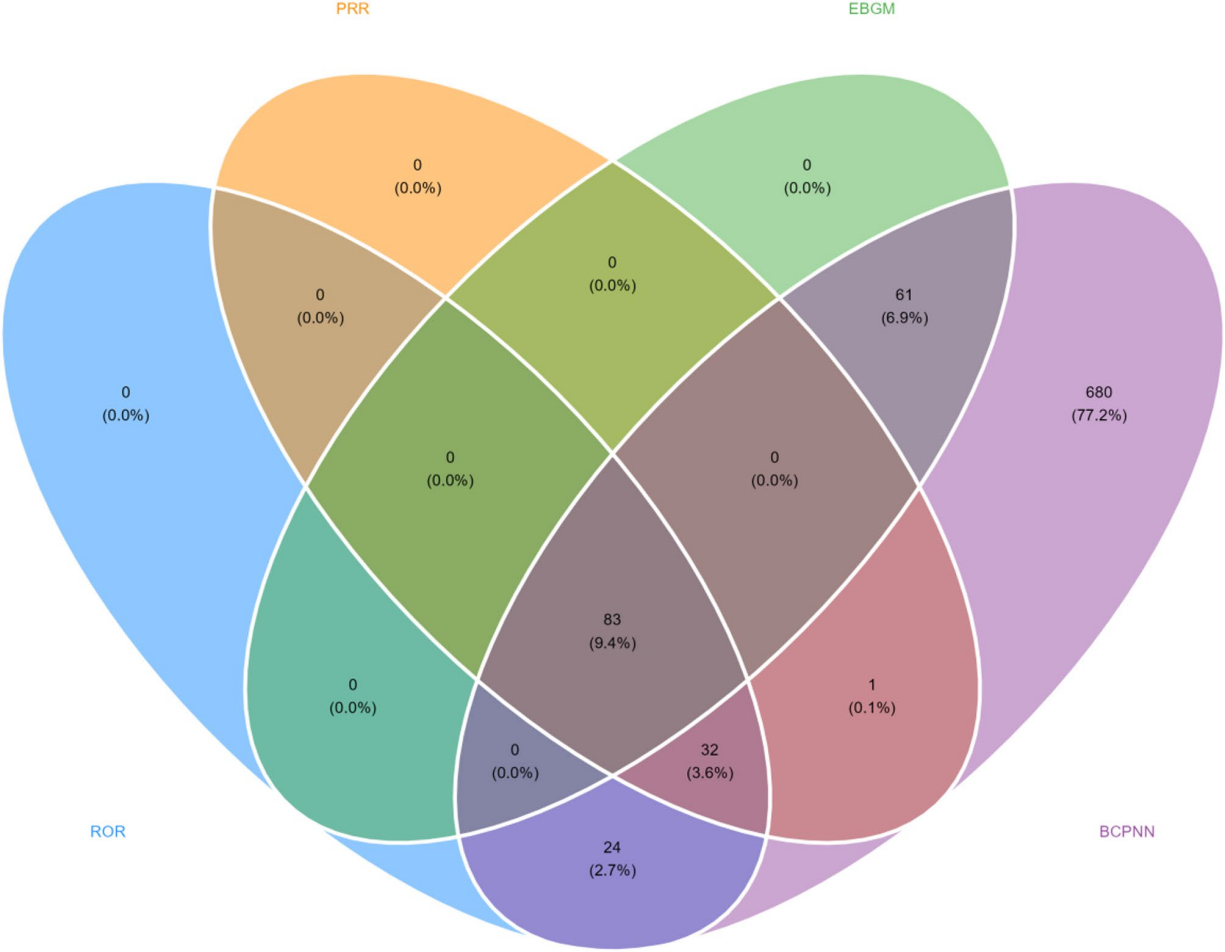
Signals identified by the four algorithms (ROR, PRR, BPCNN, and EBGM)

**Fig. 3:**
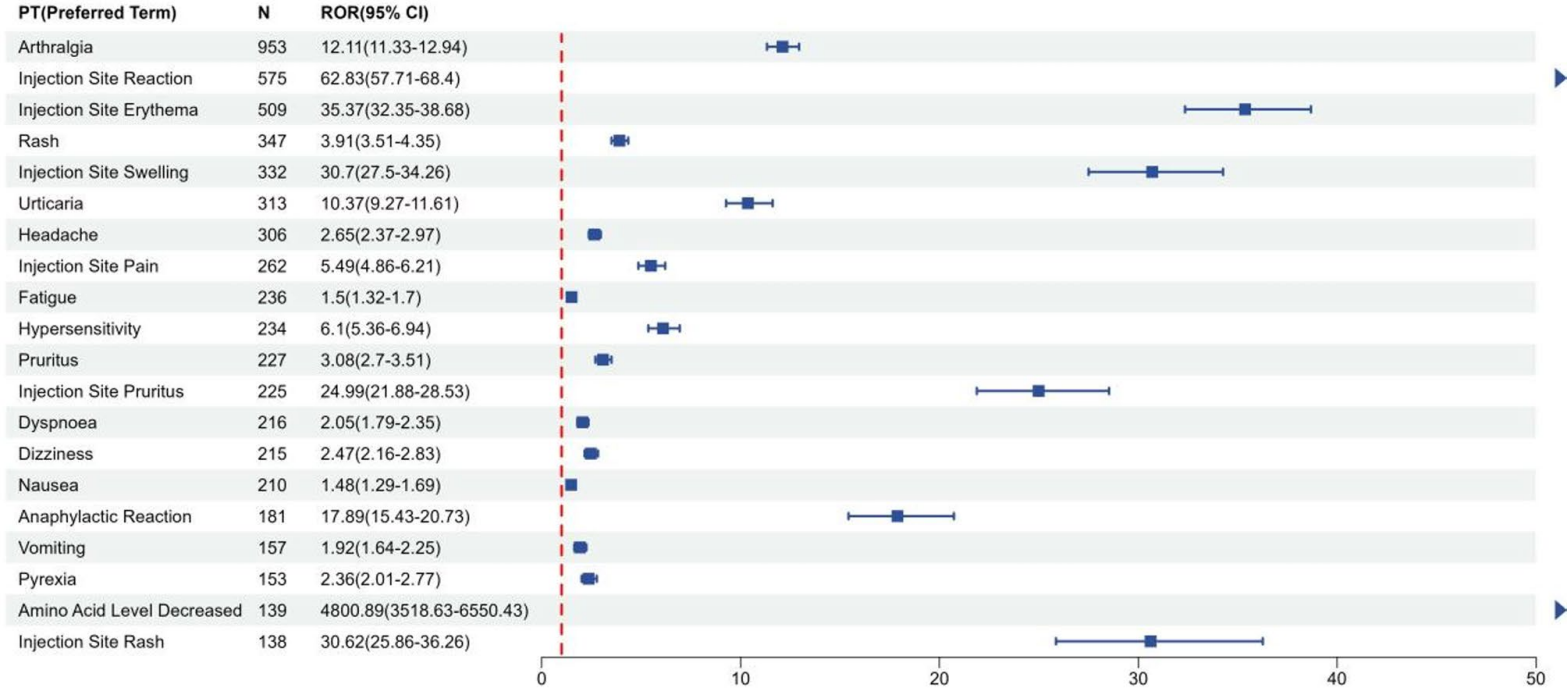
The top 20 AEs of Pegvaliase at preferred terms (PTs) level ranked by Case Numbers in FDA Adverse Event Reporting System (FAERS)

**Fig. 4:**
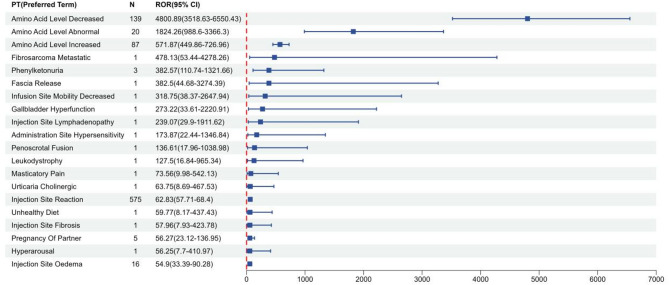
The top 20 AEs of Pegvaliase at preferred terms (PTs) level ranked by signal strength in FDA Adverse Event Reporting System (FAERS)

Signal strength and pegvaliase reports at the System Organ Class (SOC) level, ranked by the frequency of adverse events, were shown in Table 4. According to statistics, we discovered that 27 SOCs were all targeted by pegvaliase-induced AEs. General disorders and administration site conditions (32.11%), musculoskeletal and connective tissue disorders (11.93%), skin and subcutaneous tissue disorders (11.18%), nervous system disorders (7.97%), gastrointestinal disorders (7.49%) were the five most common systems. The study used the four algorithms ROR, PRR, BPCNN and EBGM to examine the adverse reactions of the drug and to assess whether it meet pre-defined screening criteria. The four algorithms identified a total of 5,076 AEs. Figure 2 shows that a total of 83 PTs, which adhere to all four algorithms, were detected among these PTs. Ranked by the frequency (Fig. 3 and Supplementary Table 1), the top 5 PTs include arthralgia (ROR 12.11, 95%CI 11.3-12.94), injection site reaction (ROR 62.83, 95% CI 57.71–68.4), injection site erythema (ROR 35.37, 95%CI 32.35–38.68); rash (ROR 3.91, 95%CI 3.51–4.35), and injection site swelling (ROR 30.7, 95%CI 27.5-34.26). Ranked by the signal intensities (Fig. 4 and Supplementary Table 2), the top 5 PTs include amino acid level decreased (ROR 4800.89, 95%CI 3518.63-6550.43), amino acid level abnormal (ROR 1824.26, 95%CI 988.6-3366.3), amino acid level increased (ROR 571.87, 95%CI 449.86-726.96), fibrosarcoma metastatic (ROR 478.13, 95%CI 53.44-4278.26), and phenylketonuria (ROR 382.57, 95%CI 110.74-1321.66), which may be related to diseases of the patients.

**Table 4:** Number of AEs of Pegvaliase at the system organ class (SOC) level in FAERS database

SOC	*N*	proportion(%)
General Disorders And Administration Site Conditions	3959	32.11
Gastrointestinal Disorders	923	7.49
Nervous System Disorders	983	7.97
Skin And Subcutaneous Tissue Disorders	1378	11.18
Musculoskeletal And Connective Tissue Disorders	1471	11.93
Psychiatric Disorders	350	2.84
Blood And Lymphatic System Disorders	43	0.35
Respiratory, Thoracic And Mediastinal Disorders	720	5.84
Infections And Infestations	435	3.53
Eye Disorders	97	0.79
Metabolism And Nutrition Disorders	108	0.88
Investigations	511	4.14
Immune System Disorders	468	3.8
Vascular Disorders	142	1.15
Injury, Poisoning And Procedural Complications	376	3.05
Cardiac Disorders	84	0.68
Pregnancy, Puerperium And Perinatal Conditions	39	0.32
Reproductive System And Breast Disorders	30	0.24
Social Circumstances	12	0.1
Product Issues	24	0.19
Renal And Urinary Disorders	44	0.36
Hepatobiliary Disorders	16	0.13
Surgical And Medical Procedures	30	0.24
Congenital, Familial And Genetic Disorders	9	0.07
Ear And Labyrinth Disorders	40	0.32
Endocrine Disorders	10	0.08
Neoplasms Benign, Malignant And Unspecified (Incl Cysts And Polyps)	27	0.22

### Subgroup analyses

We use subgroup analysis for additional research to thoroughly examine the data outcomes. Referring to Supplementary Fig. 1, the male’s top 5 PTs according to signal intensities were arthralgia (ROR 15.96, 95%CI 14.13–18.03), injection site reaction (ROR 80.48, 95%CI 68.16–95.03), injection site erythema (ROR 61.51, 95%CI 51.98–72.78), injection site swelling (ROR 44.08, 95%CI 36.06–53.87), and rash (ROR 3.97, 95%CI 3.22–4.9). The female’s top 5 PTs according to signal intensities were arthralgia (ROR 9.9, 95%CI 8.92–10.99), injection site erythema (ROR 30.18, 95%CI 26.53–34.34); injection site reaction (ROR 52.73, 95%CI 46.27–60.1), rash (ROR 4.08, 95%CI 3.49–4.77), and headache (ROR 2.57, 95%CI 2.18–3.01). We could find that the adverse reactions to pegvaliase were mostly the same in people of different sexes.

We also examined the ranking of AEs and age based on signal intensity (Supplementary Fig. 2). For those under the age of 18, the top 5 PTs were as follows: arthralgia (ROR 34.45, 95%CI 22.64–52.42); urticaria (ROR 11.15, 95%CI 6.39–19.47); injection site reaction (ROR 62.62, 95%CI 34.17-114.78); injection site swelling (ROR 44, 95%CI 24.03–80.57); vomiting (ROR2.58, 95%CI 1.33–5.01). Regarding the ≥ 18, <65 age range, the top 5 PTs were as follows: arthralgia (ROR 9.98, 95%CI 9.01–11.05); injection site erythema (ROR 15.81, 95%CI 13.88–18.02); injection site reaction (ROR 30.44, 95%CI 26.71–34.7); headache (ROR 2.2, 95%CI 1.88–2.58); injection site swelling (ROR 17.59, 95%CI 14.99–20.63). Regarding the age bracket of ≥ 65, the top 5 PTs were as follows: peripheral swelling (ROR 38.98, 95%CI 8.96-169.55); injection site reaction (ROR 136.24, 95%CI 18.13-1023.96); abdominal distension (ROR 36.07, 95%CI 4.8-271.03); headache (ROR 7.82, 95%CI 1.04–58.77); fatigue (ROR 4.67, 95%CI 0.62–35.13). We could find that the adverse reactions to pegvaliase were mostly the same in people of different ages, occurred mainly between the ages of 18–65 years.

Additionally, we explored the relationship between reporters and AEs ranked by the signal intensities (Supplementary Fig. 3). The health professional’s top 5 PTs according to signal intensities were arthralgia (ROR 10.73, 95%CI 8.06–14.28), anaphylactic reaction (ROR 28.08, 95%CI 19.5-40.44), rash (ROR 2.9, 95%CI 1.88–4.47), nausea (ROR 2.28, 95%CI 1.48–3.51), and headache (ROR 3.36, 95%CI 2.18–5.18). The consumer’s top 5 PTs according to signal intensities were arthralgia (ROR 11.53, 95%CI 10.72–12.4), injection site reaction (ROR 70.08, 95%CI 63.97–76.78), injection site erythema (ROR 31.79, 95%CI 28.86–35.03), injection site swelling (ROR 29.09, 95%CI 25.77–32.83), and rash (ROR 3.93, 95%CI 3.49–4.44). We could find that the adverse reactions to pegvaliase were mostly the same in people of different reporters.

**Fig. 5:**
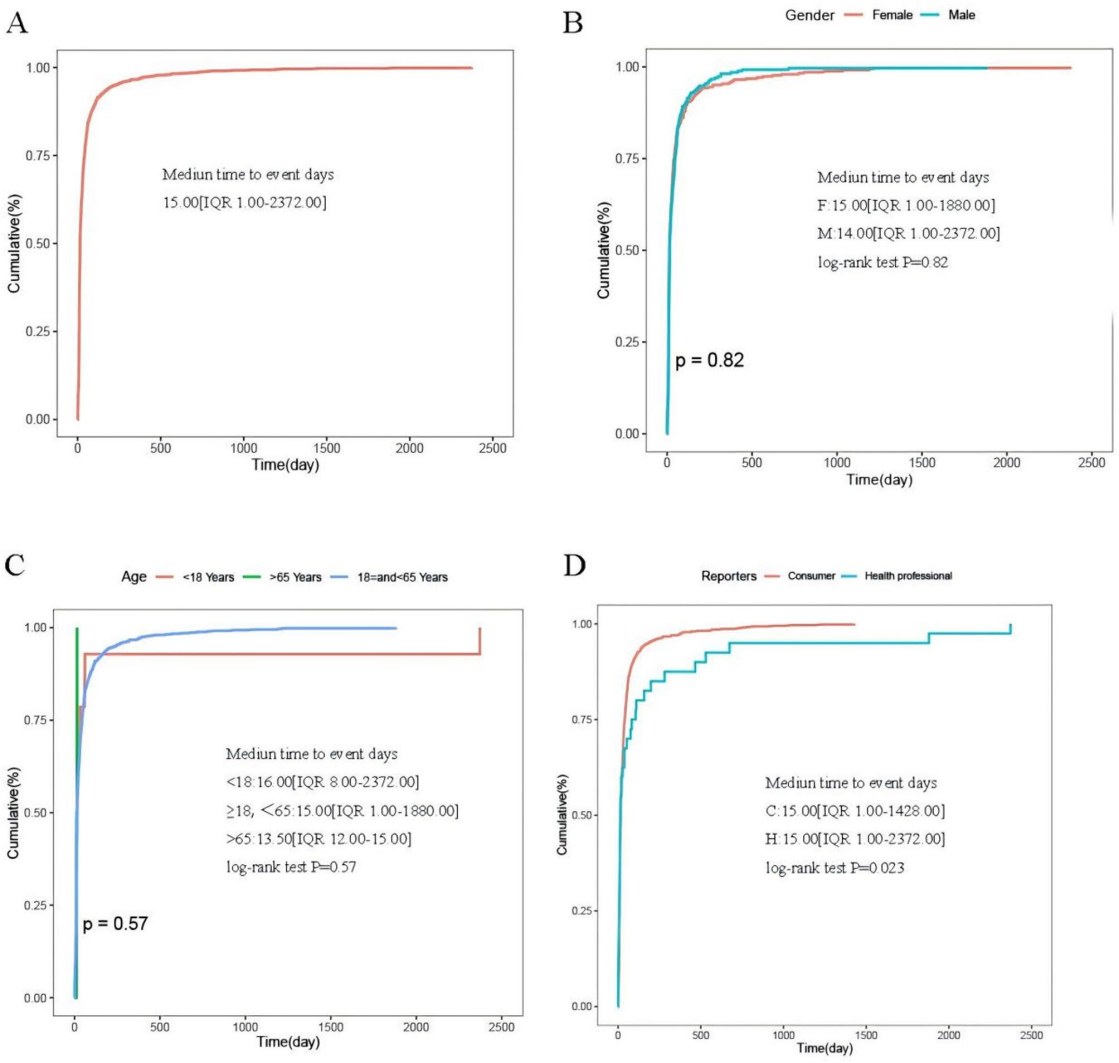
Subgroup analyses of Time to onset

We also explored the impact of different genders, ages and reporters on the time to onset time (Fig. 5). Based on the analysis provided, the median onset time of pegvaliase was 15.00 days (with a range of 1.00-2372.00 days). In our analysis of subgroups, it was found that females had a median time to events of 15.00 days (IQR 1.00-1880.00), while males had a median time of 14.00 days (IQR 1.00-2372.00). The median onset time of pegvaliase for individuals under 18 years old, ≧18 and < 65, and ≧ 65 was 16.00 (IQR 8.00-2372.00), 15.00 (IQR 1.00-1880.00), and 13.50 (IQR 12.00–15.00) respectively. The results showed that the customers had a median time to event of 15.00 days (IQR 1.00-1428.00), whereas the health professionals had a median time to incident of 15.00 days (IQR 1.00-2372.00).

## Discussion

Pegvaliase is a new enzyme substitution therapy that decreases blood Phe without the need of PAH or its BH4 cofactor [1]. The standard therapy of PKU includes a Phe-restricted diet plus medical nutrition therapy (MNT), with or without sapropterin dihydrochloride, to reduce blood Phe levels. Compared with standard therapy, pegvaliase induces a substantial, progressive, and sustained decrease in blood Phe levels - to a much greater extent than sapropterin combined with MNT or MNT alone - which is expected to improve long-term outcomes in patients with PKU [25]. A series of studies have confirmed that pegvaliase has more significant efficacy of pegvaliase in the treatment of PKU. Nevertheless, at the same time, the management of its AEs still cannot be ignored. Previous research on AEs to Pegvaliase primarily relied heavily on clinical studies and case reports. Nevertheless, strict trial designs and stringent inclusion criteria might have led to the underreporting of rare AEs, resulting in incomplete drug safety information. Our study systematically evaluated the adverse reactions related to pegvaliase by in-depth analysis of the FAERS database from 2018 Q3 to 2023 Q2. In this process, this study not only confirmed some existing safety information, but also revealed new potential risks. This provides more comprehensive and accurate data support for medical practice and medication decisions.

The results of our study show that the number of patients with adverse events is slightly more in women than in men. This could mean that women were more likely to report these side effects or that they were more vulnerable to pegvaliase’s negative effects. The most common age group in these reports is 18 to 45 years old, most likely because this is the age group that uses the drug the most. Pegvaliase is authorized as an enzyme-substitution therapy for adults (US) or patients aged ≥ 16 years (Europe) with PKU and uncontrolled blood Phe concentrations > 600µmol/L on current management. The safety and effectiveness of pegvaliase in populations under the age of 16 are currently the subject of very limited information. In one study, a 12-year-old girl was closely monitored while using Pegvaliase, and the results were excellent and her safety profile was good [26]. With the advancement of medical research, the drug is expected to be used in patients under the age of 16. To validate these results, nevertheless, greater sample sizes and sound methodology are needed. Reports of adverse reactions increased each year during the first three years of the drug’s availability, which we believe may be related to a lack of awareness of the drug among healthcare professionals and patients, or perhaps an increase in the use of the drug. With the introduction of guidelines and prevention of adverse effects by healthcare professionals and patients, the number of reported AEs was decreasing year by year. Furthermore, we discovered that 83% of the reporters were not medical professionals like doctors, pharmacists, or nurses, but rather consumers. Because the FAERS is a self-reporting system, not everyone is willing to record AEs, which could result in variations in the reporting population’s characteristics. Almost all reports are from the United States, which may be due to the fact that pegvaliase was initially marketed in the United States or due to limitations in the FAERS database. Notably, a total of 199 cases of serious outcomes, such as death, disability requiring hospitalization or life-threatening conditions, were recorded in the outcome section. But unfortunately, the FAERS database lacked sufficient information to assess the possible link between pegvaliase use and serious outcomes. The timing of adverse reactions was mainly in the first 6 months after dosing, which is consistent with the results of clinical trials. It is worth noting that roughly half of the data does not include precise timeframes for adverse events to occur, which restricts our comprehension of how adverse events occur at various drug periods. Accurate medication time data is necessary for future study, which examines how the medicine reacts differently at various medication times.

Our study showed that common PTs of pegvaliase in terms of frequency include arthralgia, injection site reaction, injection site erythema, rash, injection site swelling, et al., which are were generally consistent with the drug inserts and clinical trial results [13, 15, 19, 27]. Final results from the long-term Phase 3 clinical trial program show that the most commonly reported AEs of pegvaliase were arthralgia, injection-site reaction (ISR), injection-site erythema, and headache, which occurred most frequently during the first 6 months of therapy and most were mild or moderate in severity. Furthermore, of the 261 participants treated with pegvaliase, a total of 101 patients discontinued the trial, including 69.0% of patients (54 in the PRISM-1 study, 46 in the PRISM-2 study, and 1 in the 165–304 study) due to adverse events occurring during the first six months of treatment [28]. Pegvaliase is a bacterially derived protein, patients produce anti-drug antibodies to pegvaliase and immunologic adverse events (AEs) are expected [15]. A common worry with biologic therapies is immunogenicity, especially when it comes to nonhuman enzymes. HAEs were identified using a modified hypersensitivity SMQ, which included the additional preferred term AEs of arthralgia, arthritis, eye inflammation, eye irritation, eye pain, joint stiffness, joint swelling, pyrexia, blurred vision and polyarthritis, and the broad algorithmic anaphylactic reaction SMQ. The results of studies demonstrate that development of HAEs was temporally associated with immune response rather than pegvaliase plasma levels. Patients without anaphylaxis or SAEs and those with fewer HAEs throughout the PRISM-1 induction and titration phases generally had lower ADA titers and greater pegvaliase Ctrough. Patients with higher pegvaliase trough plasma concentrations typically had lower ADA titers and a more favorable tolerability profile. When administered doses of pegvaliase were low and both circulating immune complex concentrations and HAEs were peaking during induction/titration. Over time, the frequency of HAEs decreased as circulating immune complex concentrations decreased [29]. Therefore, a protocol for induction, titration, and maintenance (I/T/M) dosage is followed when administering pegvaliase in order to minimise the incidence of serious adverse events [15]. Beginning with low-dose injections for four weeks (an induction period of 2.5 mg/week), the dosage is progressively increased to a maintenance dose of up to 60 mg daily, contingent upon patient tolerance and pharmacological efficacy. The duration of time needed to achieve efficacy on pegvaliase and the dosage needed vary due to individual immune response [30]. The maximum dose for pegvaliase prescribed by the FDA at this time is 60 mg per day. According to studies, increasing the dosage of pegvaliase to 80 mg per day may be effective for patients who don’t respond well at lower doses, and pegvaliase dosing must be personalized to achieve therapeutic goals [31].

From the results of the study, we can find that Amino Acid Level Decreased, Amino Acid Level Abnormal, and Amino Acid Level Increased ranked high on the ROR, and that there is a mechanistic link between fluctuations in amino acid levels (especially Phe) and the enzymatic activity of pegvaliase. As a phenylalanine metabolizing enzyme, pegvaliase directly regulates the concentration of Phe, and the transient amino acid changes may reflect efficacy rather than toxicity, as tight control of Phe remains the primary therapeutic goal in PKU. We believe that the clinical significance depends on the magnitude and duration of the deviation: hypophenylalaninemia (blood Phe < 30 µmol/L) is likely to result in proteolytic metabolism; persistent elevation of blood Phe above 360 µmol/L may result in neurocognitive deficits. the lack of quantitative data to assess severity in FAERS cases is a shortcoming. Additionally, signal strength analysis expose several potential PTs not documented in Pegvaliase’s instructions, including Fibrosarcoma Metastatic, Phenylketonuria, Fascia Release, and Gallbladder Hyperfunction. A search was conducted using databases such as PubMed with the search terms Pegvaliase, Fibrosarcoma Metastatic, phenylketonuria, Fascia Release, and Gallbladder Hyperfunction. No case reports were found in the search results. These PTs might, in the context of clinical practice, be false positive data or infrequent, undetected ADR signals. Therefore, it is important for clinicians to pay attention to and validate these potential adverse drug reactions (ADRs) during the use of Pegvaliase, even though they are not listed in the drug manual [32]. To improve drug tolerability, especially during the initial treatment phase when HAEs are most frequent, specific measures can be implemented. One way to minimize the risks associated with initial treatment is by following I/T/M dosing regimens [32]. Additionally, until patients and observers are able to confidently manage dosing and recognize HAE symptoms, initial pegvaliase doses should be administered under the supervision of a healthcare practitioner. All patients taking pegvaliase should have access to autoinjectable epinephrine in order to treat any potential acute systemic hypersensitivity reaction (ASHR), and they should also receive education on how to identify ASHR symptoms and manage them [33, 34]. Patients who experience episodes of arthralgia can use drugs like acetaminophen to control their symptoms. It is advised that patients switch up their injection sites to avoid injection site reactions. Topical H1-antihistamines, topical steroids, or cold compresses can all be used to treat these symptoms [19, 32]. Another therapeutic strategy for severe and recurring HAEs could be 13-step desensitization, which would allow the medication to continue to be successful [35]. Dose reduction is often more beneficial than stopping treatment after a HAE because it preserves the “desensitizing” impact of ongoing antigen exposure. Based on the severity of the occurrence, a temporary dose reduction might be required. Reversing the dosage schedule by one or two steps may be necessary for this.

## Limitations

This study provides valuable insights into the safety profile of pegvaliase through the use of the FAERS database, offering a real-world perspective on post-market AEs.Nonetheless, a number of restrictions must be noted. First, the FAERS database relies on spontaneous reporting, which is prone to incomplete data, underreporting, and reporting bias. This may cause some adverse events to be overreported while others are underreported, especially in regions or countries with weaker reporting systems. Furthermore, the absence of long-term follow-up data in the FAERS database restricts our capacity to evaluate the persistence and long-term effects of AEs linked to pegvaliase. Long-term medication safety is a crucial factor in inherited diseases, thus this is especially crucial. Lastly, the FAERS database cannot establish a causal relationship between medications and adverse events, it can only identify associational signals. Confounding factors such as patient individual differences, comorbidities, and medication history may influence the occurrence of adverse events. Therefore, results interpretation must be more cautious to avoid over inferring causality.

## Conclusion

This study retrospectively analyzed the AEs data of pegvaliase in the FAERS database, and further analyzed the data using a variety of algorithms. The data were also analyzed in subgroups at different levels such as gender, age, reporters and time to onset. This study obtained more authentic and complete drug safety information, which can provide reference for clinical drug use and promote rational clinical drug use. In this process, this study not only confirmed some existing safety information, but also revealed new potential risks. Pegvaliase is one of the few medications available for the treatment of PKU, so it is critical to educate patients and their caregivers about pegvaliase treatment, dosing, and the risks of AEs, especially during the first 6 months of treatment.

## Electronic supplementary material

Below is the link to the electronic supplementary material.

Supplementary Material 1

## Data Availability

Data are available on the FAERS database.
