# Supplementary material for: Toxicity spectrum of pegvaliase: A pharmacovigilance analysis using the FAERS database
Source: Orphanet J Rare Dis. 2025 Jun 20;20:315. doi: 10.1186/s13023-025-03864-4 (PMC12181874; doi:10.1186/s13023-025-03864-4)

**Supplementary materials**

**Supplementary Table 1** The top 50 AEs of Pegvaliase at preferred terms (PTs) level ranked by Case Numbers in FDA Adverse Event Reporting System (FAERS)

**Supplementary Table 2** The top 50 AEs of Pegvaliase at preferred terms (PTs) level ranked by signal strength in FDA Adverse Event Reporting System (FAERS)

**Supplementary Figure 1** The top 40 AEs of Pegvaliase to females and males at preferred terms (PTs) level ranked by signal strength in FDA Adverse Event Reporting System (FAERS)

**Supplementary Figure 2** The top AEs of Pegvaliase to different age groups at preferred terms (PTs) level ranked by signal strength in FDA Adverse Event Reporting System (FAERS)

**Supplementary Figure 3** The top 40 AEs of Pegvaliase to different reporters at preferred terms (PTs) level ranked by signal strength in FDA Adverse Event Reporting System (FAERS)

**Supplementary Table 1** The top 50 AEs of Pegvaliase at preferred terms (PTs) level ranked by Case Numbers in FDA Adverse Event Reporting System (FAERS)

| PT | N | ROR(95% CI) |
| --- | --- | --- |
| Arthralgia | 953 | 12.11(11.33-12.94) |
| Injection Site Reaction | 575 | 62.83(57.71-68.4) |
| Injection Site Erythema | 509 | 35.37(32.35-38.68) |
| Rash | 347 | 3.91(3.51-4.35) |
| Injection Site Swelling | 332 | 30.7(27.5-34.26) |
| Urticaria | 313 | 10.37(9.27-11.61) |
| Headache | 306 | 2.65(2.37-2.97) |
| Injection Site Pain | 262 | 5.49(4.86-6.21) |
| Fatigue | 236 | 1.5(1.32-1.7) |
| Hypersensitivity | 234 | 6.1(5.36-6.94) |
| Pruritus | 227 | 3.08(2.7-3.51) |
| Injection Site Pruritus | 225 | 24.99(21.88-28.53) |
| Dyspnoea | 216 | 2.05(1.79-2.35) |
| Dizziness | 215 | 2.47(2.16-2.83) |
| Nausea | 210 | 1.48(1.29-1.69) |
| Anaphylactic Reaction | 181 | 17.89(15.43-20.73) |
| Vomiting | 157 | 1.92(1.64-2.25) |
| Pyrexia | 153 | 2.36(2.01-2.77) |
| Amino Acid Level Decreased | 139 | 4800.89(3518.63-6550.43) |
| Injection Site Rash | 138 | 30.62(25.86-36.26) |
| Pain | 135 | 0.94(0.79-1.11) |
| Injection Site Bruising | 133 | 10.91(9.2-12.96) |
| Chest Discomfort | 117 | 6.2(5.17-7.45) |
| Alopecia | 115 | 2.48(2.06-2.98) |
| Chest Pain | 111 | 3.82(3.17-4.61) |
| Erythema | 111 | 2.31(1.92-2.79) |
| Chills | 108 | 5.06(4.19-6.12) |
| Throat Tightness | 106 | 23.29(19.21-28.23) |
| Diarrhoea | 106 | 0.81(0.67-0.98) |
| Pain In Extremity | 99 | 1.82(1.5-2.22) |
| Anxiety | 98 | 1.82(1.49-2.22) |
| Back Pain | 90 | 2.09(1.7-2.57) |
| Amino Acid Level Increased | 87 | 571.87(449.86-726.96) |
| Cough | 86 | 1.49(1.21-1.85) |
| Abdominal Pain Upper | 76 | 2.01(1.6-2.52) |
| Flushing | 69 | 4.77(3.76-6.04) |
| Peripheral Swelling | 68 | 1.75(1.38-2.22) |
| Injection Site Urticaria | 65 | 16.83(13.18-21.5) |
| Myalgia | 61 | 2.16(1.68-2.77) |
| Injection Site Induration | 61 | 35.31(27.39-45.51) |
| Heart Rate Increased | 61 | 3.37(2.62-4.33) |
| Swelling Face | 60 | 5.33(4.13-6.87) |
| Injection Site Warmth | 57 | 27.09(20.84-35.2) |
| Abdominal Pain | 57 | 1.37(1.05-1.77) |
| Oropharyngeal Pain | 55 | 2.95(2.27-3.85) |
| Nasopharyngitis | 54 | 1.47(1.13-1.92) |
| Paraesthesia | 53 | 1.91(1.46-2.51) |
| Joint Swelling | 48 | 1.77(1.33-2.35) |
| Decreased Appetite | 47 | 1.03(0.77-1.37) |
| Migraine | 46 | 2.37(1.78-3.17) |

**Supplementary Table 2** The top 50 AEs of Pegvaliase at preferred terms (PTs) level ranked by signal strength in FDA Adverse Event Reporting System (FAERS)

| PT | N | ROR(95% CI) |
| --- | --- | --- |
| Amino Acid Level Decreased | 139 | 4800.89(3518.63-6550.43) |
| Amino Acid Level Abnormal | 20 | 1824.26(988.6-3366.3) |
| Amino Acid Level Increased | 87 | 571.87(449.86-726.96) |
| Fibrosarcoma Metastatic | 1 | 478.13(53.44-4278.26) |
| Phenylketonuria | 3 | 382.57(110.74-1321.66) |
| Fascia Release | 1 | 382.5(44.68-3274.39) |
| Infusion Site Mobility Decreased | 1 | 318.75(38.37-2647.94) |
| Gallbladder Hyperfunction | 1 | 273.22(33.61-2220.91) |
| Injection Site Lymphadenopathy | 1 | 239.07(29.9-1911.62) |
| Administration Site Hypersensitivity | 1 | 173.87(22.44-1346.84) |
| Penoscrotal Fusion | 1 | 136.61(17.96-1038.98) |
| Leukodystrophy | 1 | 127.5(16.84-965.34) |
| Masticatory Pain | 1 | 73.56(9.98-542.13) |
| Urticaria Cholinergic | 1 | 63.75(8.69-467.53) |
| Injection Site Reaction | 575 | 62.83(57.71-68.4) |
| Unhealthy Diet | 1 | 59.77(8.17-437.43) |
| Injection Site Fibrosis | 1 | 57.96(7.93-423.78) |
| Pregnancy Of Partner | 5 | 56.27(23.12-136.95) |
| Hyperarousal | 1 | 56.25(7.7-410.97) |
| Injection Site Oedema | 16 | 54.9(33.39-90.28) |
| Injection Site Scar | 25 | 47.29(31.79-70.35) |
| Idiopathic Generalised Epilepsy | 1 | 46.65(6.42-339.15) |
| Tongue Rough | 1 | 46.65(6.42-339.15) |
| Tracheal Oedema | 1 | 43.47(5.99-315.52) |
| Complement Factor C4 Decreased | 1 | 36.09(4.99-260.96) |
| Injection Site Hypertrophy | 2 | 35.42(8.74-143.46) |
| Laparoscopy | 1 | 35.42(4.9-256.04) |
| Injection Site Erythema | 509 | 35.37(32.35-38.68) |
| Injection Site Induration | 61 | 35.31(27.39-45.51) |
| Injection Site Swelling | 332 | 30.7(27.5-34.26) |
| Injection Site Rash | 138 | 30.62(25.86-36.26) |
| Injection Site Warmth | 57 | 27.09(20.84-35.2) |
| Complement Factor C3 Decreased | 1 | 26.56(3.69-191.17) |
| Genital Contusion | 1 | 26.56(3.69-191.17) |
| Circumoral Oedema | 1 | 25.5(3.55-183.42) |
| Injection Site Pruritus | 225 | 24.99(21.88-28.53) |
| Nodular Rash | 1 | 24.84(3.45-178.6) |
| Throat Tightness | 106 | 23.29(19.21-28.23) |
| Inguinal Mass | 1 | 23.04(3.21-165.54) |
| Drug Titration | 1 | 23.04(3.21-165.54) |
| B-Cell Small Lymphocytic Lymphoma | 1 | 22.77(3.17-163.54) |
| Epileptic Aura | 1 | 22.24(3.1-159.7) |
| Injection Site Papule | 17 | 22.07(13.68-35.61) |
| Verbal Abuse | 1 | 21.98(3.06-157.84) |
| Visual Brightness | 1 | 21.73(3.03-156.03) |
| Emphysematous Cystitis | 1 | 21.73(3.03-156.03) |
| Injection Site Laceration | 2 | 20.9(5.19-84.23) |
| Anorectal Operation | 1 | 20.79(2.9-149.17) |
| Vaccination Site Swelling | 1 | 20.56(2.87-147.55) |
| Mineral Deficiency | 1 | 20.56(2.87-147.55) |

**Supplementary Figure 1** The top 40 AEs of Pegvaliase to females and males at preferred terms (PTs) level ranked by signal strength in FDA Adverse Event Reporting System (FAERS)


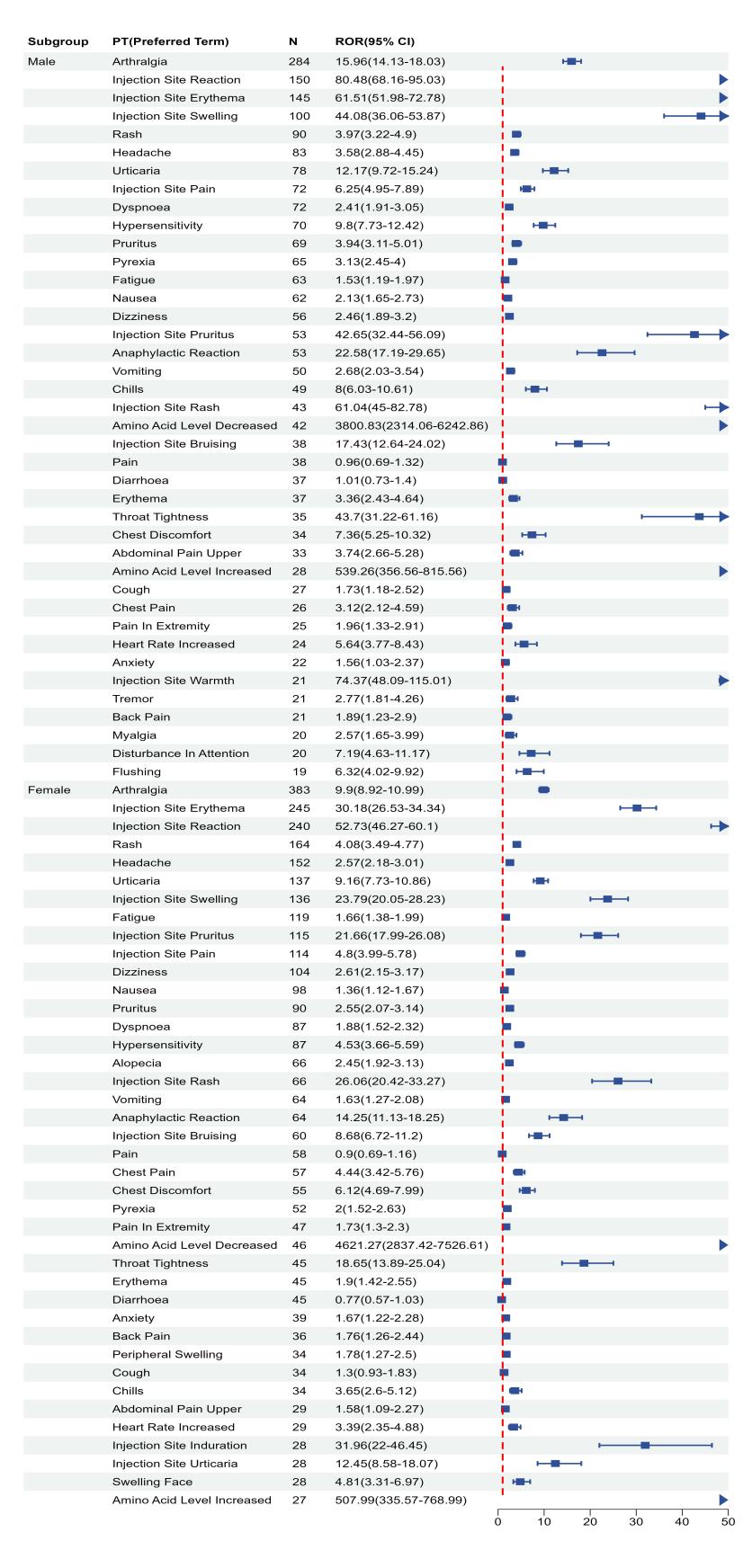


**Supplementary Figure 2** The top AEs of Pegvaliase to different age groups at preferred terms (PTs) level ranked by signal strength in FDA Adverse Event Reporting System (FAERS)


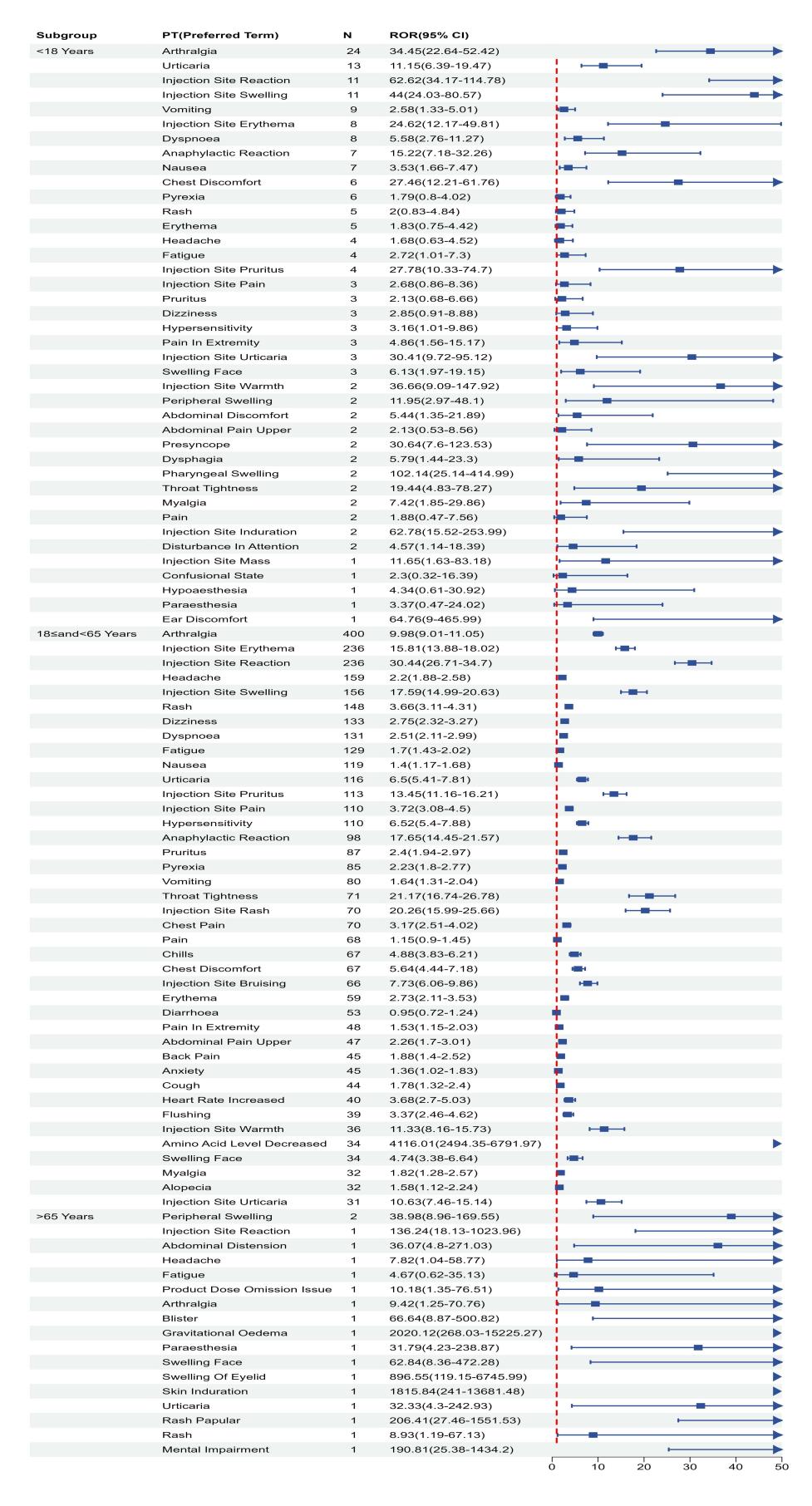


**Supplementary Figure 3** The top 40 AEs of Pegvaliase to different reporters at preferred terms (PTs) level ranked by signal strength in FDA Adverse Event Reporting System (FAERS)


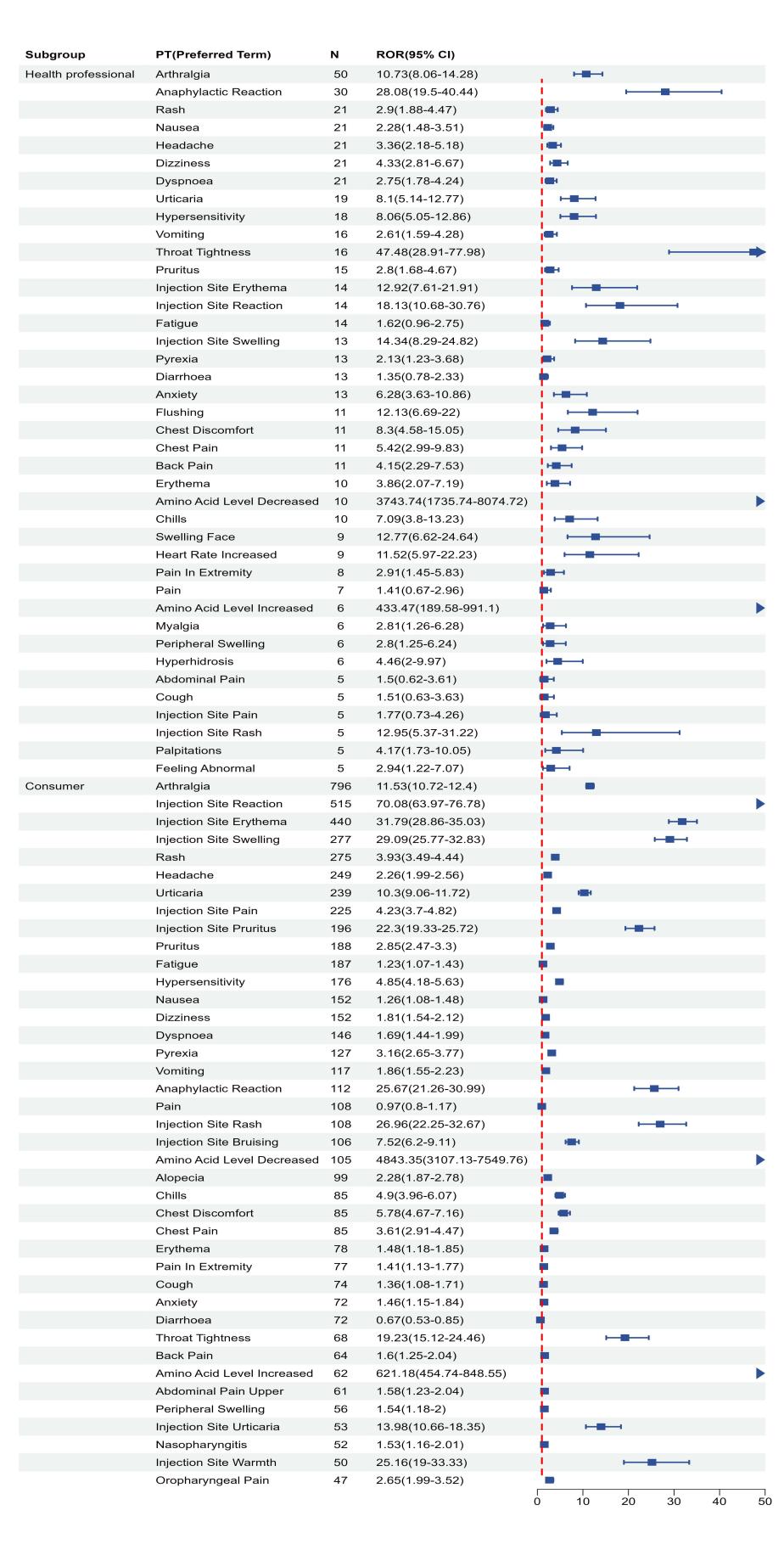

Supplement: Supplementary file 1 — Supplementary Material 1 [file 13023_2025_3864_MOESM1_ESM.docx]
